# Supplementary material for: The CXCL10/CXCR3 Pathway Contributes to the Synergy of Thermal Ablation and PD-1 Blockade Therapy against Tumors
Source: Cancers (Basel). 2023 Feb 23;15(5):1427. doi: 10.3390/cancers15051427 (PMC10000434; doi:10.3390/cancers15051427)
Supplement: Supplementary file 1 [file cancers-15-01427-s001.zip › cancers-2175323-supplementary.docx]

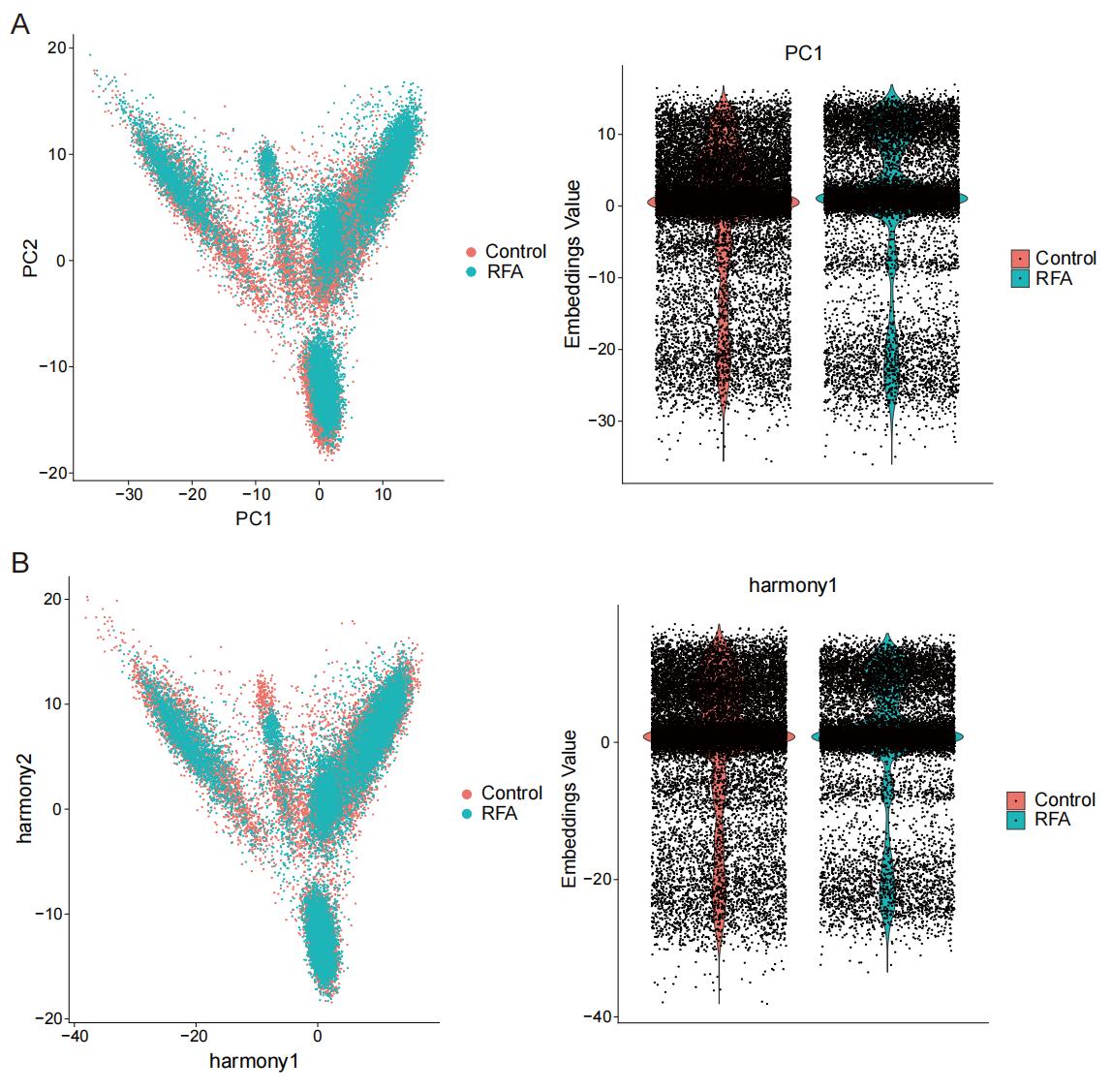


**Figure S1.** **Immune cells on the non-RFA side of the PDAC mouse model.** **A**-**B.** scRNA-seq data from 26084 cells were obtained before (**A**) and after (**B**) batch removal.
